# A process evaluation of the PLAN-A intervention (Peer-Led physical Activity iNtervention for Adolescent girls)

**DOI:** 10.1186/s12889-019-7545-z

**Published:** 2019-09-02

**Authors:** Simon J. Sebire, Kathryn Banfield, Russell Jago, Mark J. Edwards, Rona Campbell, Ruth Kipping, Peter S. Blair, Bryar Kadir, Kirsty Garfield, Joe Matthews, Ronan A. Lyons, William Hollingworth

**Affiliations:** 1 Centre for Exercise, Nutrition and Health Sciences, School for Policy Studies, University of Bristol, Bristol, UK; 2 National Institute for Health Research (NIHR) Collaboration for Leadership in Applied Health Research and Care West (CLAHRC West), University Hospitals Bristol NHS Foundation Trust, Bristol, UK; 3 Department of Population Health Sciences, Bristol Medical School, University of Bristol, Bristol, UK; 4 Centre for the Development and Evaluation of Complex Interventions for Public Health Improvement (DECIPHer) School of Social Sciences, Cardiff University, Cardiff, UK; 5 Bristol Randomised Trials Collaboration, University of Bristol, Bristol, UK; 6 Farr Institute, Swansea University Medical School, Swansea, UK

**Keywords:** Physical activity, Intervention, Adolescents, Process evaluation, School

## Abstract

**Background:**

Few adolescent girls engage in enough physical activity (PA) to meet recommendations and there is a need for new interventions to increase girls PA. We have previously published the results of the PLAN-A cluster randomised feasibility trial which was a peer-led school-based PA intervention, showing that the intervention was feasible and held promise to increase the PA of girls aged 12–13 years. In PLAN-A, pupils nominated by their peers as influential attend training to teach them how to influence, promote and normalise physical activity amongst their peer-group. This paper reports the results of the process evaluation of the PLAN-A feasibility study, specifically focussing on acceptability to key stakeholders, intervention fidelity, receipt/experiences and perceived effect and suggested intervention refinements before proceeding to a definitive RCT.

**Methods:**

A mixed-methods process evaluation triangulated data from qualitative focus groups and interviews with peer-supporter and non peer-supporter pupils (*N* = 52), parents (*N* = 12), teachers (*N* = 6) and intervention training deliverers (*N* = 5), quantitative questionnaires, and observations of intervention delivery. Quantitative data were analysed descriptively, and qualitative data were analysed with the Framework Method.

**Results:**

The duration, timings, content and delivery of the peer-supporter training were acceptable. There was good fidelity to the intervention manual and its underpinning theory including high fulfilment of session objectives and use of an autonomy-supportive motivational style. Peer-supporters engaged with and enjoyed the training and retained key peer-supporter messages (what counts as PA, encouragement, empathy and subtlety). Parents and teachers were supportive of the intervention and reported perceived effects including increased PA and awareness of it, improved peer relationships, and confidence. Suggested intervention refinements included increasing participatory learning, reducing technical jargon, and providing more support to overcome challenges to giving peer support.

**Conclusions:**

PLAN-A can be delivered as planned, is well-received, and appears to be effective in empowering adolescent girls to support their peer group to become more active. The refinements identified can be made within the original intervention structure, before proceeding to a definitive trial.

**Trial registration:**

ISCTRN, ISRCTN12543546, Registered on 28/7/2015.

## Background

Physical activity has positive effects on adolescent health and wellbeing [1], including reduced risk of obesity [2] and cardiovascular disease with its associated biomarkers [3–5] and improved mental health [6]. Many young people do not meet recommendations [1] of at least 60 min of moderate-to-vigorous physical activity (MVPA) per day [7, 8]. Physical activity levels decrease throughout adolescence [7, 8] and this decline starts sooner and becomes steeper for girls compared to boys [8, 9]. As girls get older, they participate less in school physical education and club sports but do not replace this with non-competitive physical activity [10]. Studies of objectively measured physical activity show that boys expend more energy in physical activity, have lower sedentary time (338.4 vs. 383.7 min/d) and spend more time in moderate (50.0 vs. 30.3 min/d) and vigorous (8.4 vs. 1.7 min/d vigorous) physical activity than girls [9]. Therefore effective physical activity interventions for girls are needed.

The current evidence base identifies that the majority of physical activity interventions for adolescents have reported null or small effects [11]. Medical Research Council Guidance [12] highlights the value of conducting detailed process evaluations of complex public health interventions across the different stages of development, from pilot to definitive trial. Process evaluations are critical in understanding the feasibility of an intervention and identifying design refinements and can contribute important information to the decision of whether to proceed to the next stage of evaluation. Process evaluations consider factors beyond effectiveness to assess intervention implementation (e.g., delivery, fidelity & implementation processes), mechanisms of impact (e.g., participant engagement, responses to the intervention, mediators & unexpected outcomes) and the influence of contextual factors which shape how the intervention is delivered and received.

We have recently reported the protocol [13] and findings [14] of a feasibility study of PLAN-A, a Peer-Led physical Activity iNtervention for Adolescent girls. The PLAN-A intervention is based on a previously effective stop-smoking intervention, A Stop Smoking in Schools Trial (ASSIST) [15], which used informal peer-diffusion to spread positive messages and develop new norms for health behaviour (for ASSIST, not smoking; for PLAN-A, being physically active). Influential girls (i.e., those who are *looked up to, good leaders, trusted* and *respected*) in Year 8 (age 12–13 years) were identified by peer nomination. The 18% of girls with most nominations were invited to be peer-supporters. Girls who consented completed a two-day peer-supporter training programme, plus one top-up (refresher/booster session) day 5 weeks later. The peer-supporter training was held out of school (i.e. in a community venue) and led by female trainers, and was designed to: (a) increase girls’ knowledge about physical activity, (b) help them identify existing interpersonal skills and develop new ones that they can use to support their peers, (c) empower them to create new norms and diffuse positive messaging amongst their peers about getting or being active. In one case, the peer-supporter training was held on a school site because the school administrators were unable to provide a chaperone. Five peer-supporter trainers with backgrounds in physical activity or youth work/drama delivered the peer-supporter training to each school group, working in pairs. They had attended a three-day train-the-trainers event led by the study team (and received a training manual, the peer-supporter training session plans and training resources) which covered the PLAN-A design and concept, delivery of each activity, and the theoretical principles of the intervention.

The peer-led intervention mechanism was based on Diffusion of Innovations theory (DOI) [16] where social influencers amongst a group of individuals can act as change agents, to bring about change in beliefs, attitudes, and ultimately behaviour. In addition to DOI, the core tenets of self-determination theory (SDT) [17, 18] were woven into the design, delivery and content of the train-the-trainers and peer-supporter training. Full details of the intervention design, the theoretical background and information on how the intervention components mapped on to SDT are presented in the protocol paper [13].

We conducted a feasibility cluster randomised controlled trial of the PLAN-A intervention in six secondary schools (four intervention, two control) involving 427 Year 8 girls. Measures of intervention feasibility and objective assessments of girls’ MVPA at baseline, 10 weeks and 5 months follow up were collected [14]. We found that school, participant, trainer and peer-supporter recruitment and retention were feasible, that data required to evaluate the intervention could be collected and that 94% of peer-supporters attended all of the training. We also identified that the intervention had the potential to affect MVPA (i.e., a 6.1 min/d difference, 95% CI = 1.43 to 10.76 min/d) between the intervention and control arms at the 5 months follow-up [14] and determined the sample size needed for a definitive (fully powered) trial. The economic analysis showed that the PLAN-A intervention had the potential to be cost effective (£2685 per school, or £37 per Year 8 girl).

This paper reports the results of a process evaluation which was embedded in the PLAN-A feasibility study with the following objectives:

1. Assess the acceptability of the intervention to key stakeholders (i.e. peer-supporters, non-peer supporter pupils, peer-supporter trainers, school teachers & parents)
2. Assess intervention delivery and fidelity, including alignment with the underpinning theories
3. Assess the receipt/experiences of the intervention by peer-supporters and non-peer-supporters
4. Describe any potential perceived intervention effect
5. Identify intervention refinements

## Methods

### Study design

We conducted a mixed-methods process evaluation involving triangulation of qualitative and quantitative data collected from key stakeholders and observation of peer-supporter training. Ethical approval was obtained from the University of Bristol’s School for Policy Studies Research and Ethics committee (Ref: SPSREC14–15.A27). Parents could opt their child out of the study. Parents of peer-supporters provided written informed consent and peer-supporters gave written informed assent. All adults involved in the research (i.e., peer-supporter trainers, teachers & parents) provided written informed consent.

### Data collection

Table 1 shows details of the participants involved in each element of the process evaluation and associated methodological information.

**Table 1:** Data collection methods and participant details

Participant group	Data collection method	Recruitment procedure	N	Details
Peer-supporters	Focus groups (mean duration = 44 mins, range = 38 to 48 mins)	Random selection	28 (7 per intervention school)	Female 12–13 years
	Post-training questionnaire (two- day and top-up)	Completed by all peer-supporters in attendance	52	
Non-peer-supporters	Focus groups (mean duration = 37 mins, range = 21 to 49 mins)	Random selection of two participants from MVPA tertiles	24 (6 per intervention school)	Female 12–13 years
Peer-supporter trainers	Interview post two-day training (mean duration = 54 mins, range = 44 to 59 mins) and top-up training (mean duration = 40 mins, range = 32 to 49 mins)	All trainers recruited	5 (Post two-day training) 4 (Post top-up training)	Mean Age = 33.80 (SD = 9.68) years Gender: 100% female Education ranged from A Levels^a^ to Higher Degree
	Post-training questionnaire (two-day and top-up)	All trainers recruited	5	
School contact	Interview mean duration = 28 mins, range = 14 to 43 mins	All school contacts interviewed	6	Five PE teachers One student support staff. Gender: *N* = 5, 83% female
Parents of peer-supporters	Interview mean duration = 25 mins, range = 20 to 34 mins	Random selection of three parents of peer-supporters per intervention school	12	Gender: *N* = 11, 92% female

^a^A-levels are a post-16 subject-based college or sixth form leaving qualification offered by educational bodies in the United Kingdom

### Intervention delivery

#### Quantitative

Trainers completed an evaluation form developed by the research team, following the two-day and top-up day training using Likert scales to rate: (a) achievement of the peer-supporter training objectives (five items covering knowledge, interpersonal skills, communication, confidence and role clarity scored 0 = Not well at all, 3 = Very well), (b) peer-supporter engagement, involvement, interest and enjoyment (all single items scored 0 = Not at all, 3 = Very), and (c) the suitability of the training arrangements (8 items covering transport to/from the venue, suitability of the training space etc., scored 0 = Poor, 5 = Excellent).

A member of the study team observed each trainer pair once delivering day one and two and once delivering the top-up day peer-supporter training, to assess intervention fidelity including meeting of training objectives, (0 = Not at all, 3 = Lots), logistics (e.g., timing of activities) and peer-supporter engagement (0 = Not at all, 3 = Very). Observation notes were made about the trainers’ delivery style, elements that worked and those that needed refinement.

#### Qualitative

Semi-structured interviews were undertaken by a member of the research team (KB) with all trainers within 2 weeks of the two-day peer-supporter training and the top-up training. Interview guides sought trainers’ opinion on the training they received, peer-supporter training content and delivery, supporting peer-supporters’ motivation, intervention refinements and delivering the training off and on the school site. All focus groups and interviews were recorded using an encrypted digital recorder (Olympus DS-3500) and audio files transcribed verbatim and anonymised.

### Intervention receipt / experiences

#### Quantitative

Peer-supporters completed an evaluation questionnaire following the two-day and top-up day training indicating their views on the content and logistics of the training, (e.g. *I understand my role as a peer-supporter)* (0 = Disagree a lot, 4 = Agree a lot), their level of enjoyment (1 = Not at all, 5 = A lot) and free-text responses about what they enjoyed and had learnt. Peer-supporters also reported the extent to which the trainers were autonomy-supportive (e.g., “*The PLAN-A trainers provided me with choices and options”*) using the five-item Sport Climate Questionnaire [19] anchored by responses ranging from 0 (‘Disagree a lot’) to 4 (‘Agree a lot’). The mean of the five items was derived to produce an autonomy-support score for each training pair.

#### Qualitative

Focus groups were conducted with a sample of seven randomly selected peer-supporters from each intervention school (average of 10% of girls in Year 8) by KB & JM at the end of the ten-week intervention period. The focus groups explored the peer-supporter training, including content, logistics and views on the trainers, and their experience of being a peer-supporter, strategies to support their peers, any challenges and successes.

Six girls from each intervention school who were non-peer-supporters were purposively selected to participate in a focus group conducted by KB & JM based on their baseline minutes of MVPA (levels of MVPA were divided into thirds and two participants randomly sampled from each third to provide a sample with diversity in levels of MVPA). The focus group guide was used to investigate participants’ awareness of PLAN-A, views on the peer-supporters and any conversations they had with them and perceived impact of the intervention.

A member of staff at each school who served as the link between the research team and the school (school contact) participated in a semi-structured interview with KB. Topics of discussion included their level of involvement in the study, data collection arrangements, the intervention implementation and their thoughts on the peer-supporter training and potential impact of PLAN-A. Control school contacts were asked for their opinions on being randomised as a control school and view on the potential of the intervention.

Parents of peer-supporters were randomly selected to take part in telephone interviews with KB or JM. The interview guide explored parents’ views on the peer-supporter training and their daughter’s role as a peer-supporter and the perceived impact of PLAN-A on their daughter. All data collection materials are available here: https://www.journalslibrary.nihr.ac.uk/programmes/phr/139016/#/

### Analysis

#### Quantitative analysis

The quantitative analysis was largely descriptive. Means and standard deviations (SD) were used to describe participant and observer ratings and the observed duration of sessions was compared to the session timetable. Open ended responses from peer-supporters were grouped into similar categories based on their content and frequencies for each category were calculated.

#### Qualitative analysis

Qualitative data were transcribed professionally. The Framework method [20] was used to analyse the qualitative data and allow comparison of the views of the five stakeholders using both deductive and inductive approaches. Analysis was performed by team members: KB, MJE, SS and JM. After all researchers familiarised themselves with the transcripts, they each independently coded one transcript per stakeholder group. Deductively, codes addressed the logistics of the intervention, acceptability, implementation, its impact on participants and fidelity to the theory (SDT). Emerging (inductive) codes were discussed between researchers. The codes from each researcher’s initial analysis were combined to create a framework that was applied to the remaining transcripts for each stakeholder. Consistency and agreement of coding was ensured by double coding, discussing new codes and discrepancies and amending the framework. Coded data were then charted to a framework matrix in NVivo and the data summarised into themes for all stakeholder groups. Themes were generated, agreed and supported by illustrative quotes. A COnsolidated criteria for Reporting Qualitative research (COREQ) checklist was completed. Free text observation data were reduced to reflect similarities and differences in terms of what was delivered and how content was delivered between trainer pairs. Results are presented in a mixed methods format which draws on both quantitative and qualitative data.

## Results

### Acceptability of the intervention

#### Train-the-trainers

The training and intervention materials were described as thorough, clear, and well explained, and that they helped prepare trainers to deliver the peer-supporter training. Two trainers suggested that the train-the-trainers event should be held closer to delivery, with some trainers using their own time to revisit the content before delivery. Time devoted to familiarisation with and practicing delivering each activity was particularly welcomed:

*[ … ] you guys both did a good job I think explaining everything to us and then because we did it we did every activity we were going to do it gave us enough time to think about it [ … ]*

*Trainer B*

#### Peer nomination and peer-supporter training

The main reason that girls wanted to be peer-supporters was to help other girls become more active.

*I like the idea of helping others become more active [ … ] it's the idea of helping my friends to do more stuff like inside or outside, physically active.*

*School 3, Peer-supporter focus group*

Stakeholders described the peer-supporters as ‘*sporty*’, ‘*confident*’, ‘*outgoing*’ and ‘*self-motivated’* and peer-supporters described feeling a sense of “*pride*”, “*privilege*” and “*achievement*” (School 4 & 6, Peer-supporter focus group) for being nominated.

In two schools it was felt that peer-supporters represented different friendship groups

*I think there was quite a few [girls] from lots of different ones [friendship groups].*

*School 2, Peer-supporter focus group*

In some cases, friendship groups and peer nomination were affected by the school year group structure, whereby Year 8 s in all intervention schools were organised in to two halves who were often taught in different classes and had limited interaction with each other.

*I'm on one side of the year so I chose the people who were on my side of the year that I knew were good at it but obviously I wouldn't know the people on the other side of the year so I didn't put [nominate] any of them.*

*School 3, Non-peer-supporter focus group*

Some students not selected to be a peer-supporter were relieved, providing some evidence that the nomination process was effective in identifying students that were sufficiently confident to be influential on the activity of their peers.

*I didn’t want to do it because I didn’t want to have to meet new people. I don’t like talking to people who I don’t know and that made me feel really awkward about doing it.*

*School 4, Non-peer-supporter focus group*

Trainers and school contacts reported that the transport, refreshments and venue for the peer-supporter training were well organised. The venue was important in supporting the engagement and energy levels of peer-supporters and three out of four venues were acceptable. The venue for one school (amongst offices with no outdoor space) was deemed unsuitable, this was changed for the top-up day. In the school that ran the training on-site, whilst the additional time afforded by not travelling off-site was advantageous, distractions were caused by other students, the peer-supporter training timing did not align with usual school-day timings (i.e., breaks / lunch), and peer-supporter commitments (i.e. music lessons) were disruptive. Trainers were supportive of in school delivery if a venue which prevented disturbances was available:

*When you’re at school you have more time and I liked that … it was nice to have longer to do the reflection and relaxation thing, I almost prefer at the school, if there was a room where nobody was going to come into.*

*Trainer B*

The training duration (i.e., 3 days) was acceptable (*“worthwhile” - School 2, parent of peer-supporter)* to parents and peer-supporters. Observations showed that the timings of the activities worked, with a small number running over. Delivering the training in a pair was considered valuable and beneficial for both the trainers and peer-supporters. It was suggested that the same trainer pair should deliver the two-day and top-up day training to students in the same school to maintain rapport.

*I guess now looking back I don’t think it’s [having a different trainer for the top-up day] ideal because they don’t know me [ … ]*

*Trainer B*

Peer-supporter enjoyment of the training was moderate-to-high and varied between schools on Day 1 and 2, but less so on the top-up day (Table 2). Enjoyment was consistently higher on Day 2 (mean ± SD = 4.24 ± 0.58) compared with Day 1 (3.66 ± 0.77) potentially because activities on these days were more interactive. Findings were in agreement with the trainers’ perception of peer-supporter enjoyment (Table 2).

*[ … ] we didn’t just learn it by sitting down and reading things, it was a lot more enjoyable in the way that we were learning it in different ways than we would in class.*

*School 3, Peer-supporter focus group*

**Table 2:** Trainer ratings of peer-supporter involvement, enjoyment, engagement and interest in their training and fulfilment of training objectives

Training	School ID								Average	
	2		3		4		6			
	Mean	SD	Mean	SD	Mean	SD	Mean	SD	Mean	SD
Peer-supporter involvement^a^										
Two-day	2.00	0.00	2.00	0.00	3.00	0.00	2.50	0.71	2.38	0.52
Top-up day	1.50	0.71	3.00	0.00	2.50	0.71	1.50	0.71	2.13	0.84
Training mean	1.75	0.35	2.50	0.71	2.75	0.35	2.00	0.71	2.26	0.18
Peer-supporter engagement^a^										
Two-day	2.00	0.00	2.50	0.71	3.00	0.00	2.50	0.71	2.50	0.54
Top-up day	1.50	0.71	3.00	0.00	2.50	0.71	1.50	0.71	2.13	0.84
Training mean	1.75	0.35	2.75	0.35	2.75	0.35	2.00	0.71	2.32	0.26
Peer-supporter enjoyment^a^										
Two-day	2.00	0.00	2.00	0.00	3.00	0.00	2.00	0.00	2.25	0.46
Top-up day	1.50	0.71	2.00	0.00	2.50	0.71	2.00	0.00	2.00	0.54
Training mean	1.75	0.35	2.00	0.00	2.75	0.35	2.00	0.00	2.13	0.18
Peer-supporter interest^a^										
Two-day	2.00	0.00	2.00	0.00	3.00	0.00	2.00	0.00	2.25	0.46
Top-up day	2.00	0.00	2.50	0.71	2.50	0.71	1.50	0.71	2.13	0.64
Training mean	2.00	0.00	2.25	0.35	2.75	0.35	1.75	0.35	2.19	0.08
Peer-supporter engagement (researcher-observed)^a^										
Two-day	–	–	2.65	0.09	2.57	0.09	2.54	0.09	2.59	0.04
Top-up day	2.40	0.71	2.75	0.39	2.75	0.39	2.54	0.71	2.52	0.17
Training mean	2.40	0.00	2.70	0.07	2.66	0.13	2.54	0.00	2.56	0.05
Fulfilment of manualised training activity objectives (observed)^b^										
Two-day	–	–	2.53	0.30	2.70	0.00	2.25	0.24	2.49	0.18
Top-up day	2.33	1.04	2.77	0.37	2.96	0.18	2.83	0.36	2.72	0.27
Training mean	2.33	0.00	2.65	0.17	2.83	0.18	2.54	0.41	2.61	0.16

^a^0 = Not at all, 1 = A little, 2 = Quite a lot, 3 = Very. - = Not observed

^b^0 = Not well at all, 3 = Very well

Interactive elements, active games, competitions, group discussions (e.g., based on inspirational videos) and working together were important sources of enjoyment:

*I think it’s about how we all worked together on all different groups coming together. We just all agreed.*

*School 2, Peer-supporter focus group*

Trainers and school contacts agreed that the training content was appropriate for Year 8 girls and successful in educating the peer-supporters about their role. However, few peer-supporters reported using the booklet and diary and some found expressing their opinion, using the role plays effectively (role playing realistic peer support situations) and understanding some terms (e.g., sedentary behaviour) challenging:

*[ … ] a lot of us struggled when we had to give our opinion on why we picked stuff because you feel like you're either going to be wrong.*

*School 3, Peer-supporter focus group*

Enjoyment levels agree with the level of observed and trainer-rated peer-supporter engagement which was moderate-high across all 3 days and consistent between schools (Table 2). Whilst trainers and school contacts described that the peer-supporters were excited and enthusiastic to attend training, some girls were described as seeing training *“as a [ … ] whole day off school” (Trainer C, top-up)*. Challenges to delivery were made when activities involved too much sitting, waiting, repetition or writing, when some peer-supporters could not report having given support to their peers (top-up day) and when there was disruptive behaviour. These findings agree with the peer-supporter involvement and engagement ratings being lower in three of four schools on the top-up training (Table 2) in addition to peer-supporters feeling that they did not learn as much new information in the top-up day as they did on day 1 and 2 (Table 3). Trainers in one school believed that the effectiveness of the training could be dampened by some unrealistic peer-supporter role play brought about by disruptive behaviour:

*We were kind of thinking “oh my gosh, if they go and talk to their friends like this, it’s not going to be encouraging them, that’s really going to put them off”*

*Trainer E*

**Table 3:** Peer-supporter reports of understanding, learning and confidence to peer support following training

Question	School ID									
	2		3		4		6		Total	
	Mean	SD	Mean	SD	Mean	SD	Mean	SD	Mean	SD
Two-day training										
I understand my role as a peer-supporter	3.92	0.29	3.76	0.44	3.82	0.41	3.91	0.30	3.84	0.37
I learned some new things about physical activity	3.83	0.39	3.24	0.90	3.91	0.30	3.64	0.51	3.61	0.67
I am confident that I can pass positive messages on to my friends about getting active	3.75	0.45	3.24	0.83	3.36	0.92	3.00	0.63	3.33	0.68
Top-up day training										
I understand my role as a peer-supporter	3.83	0.39	3.94	0.24	4.00	0.00	3.85	0.38	3.91	0.30
I learned some new things about physical activity	3.08	1.08	2.82	0.81	2.55	1.37	3.00	0.85	2.87	1.01
I am confident that I can pass positive messages on to my friends about getting active	3.25	0.87	3.29	0.59	3.45	0.69	3.54	0.66	3.38	0.69

Response scale: 0 = Disagree a lot, 1 = Disagree a little, 2 = Neither agree or disagree, 3 = Agree a little, 4 = Agree a lot

### Delivery and receipt of peer-supporter training and experience of being a peer-supporter

All training days were delivered in all intervention schools. The training objectives were fulfilled (on average “quite a lot” to “very”) on all three training days (Table 2) and this was relatively consistent within and between trainer pairings. Observation notes showed that where certain elements were not delivered as intended this was commonly due to poor explanation, not utilising the space provided, trainers not making the link between the activity and the peer-supporter role clear, or not providing enough detail or support for more challenging tasks.

Peer-supporters understood key messages in the training (e.g., broad definition of physical activity & its importance, supporting close friends, finding activities their friends enjoy, and being confident):

*Knowing that a lot of things are exercise was really reassuring, you are doing exercise, it’s just not in some other ways.*

*School 4, Peer-supporter focus group*

*Just talking to close friends and not having to be like talking to anyone and feeling confident in ourselves even if we do say the wrong thing our close friends will know what we mean [ … ]*

*School 3, Peer-supporter focus group*

Following training, peer-supporters understood their role and were confident to support their friends (Table 3). However, peer-supporters also commonly cited needing more help with ‘Confidence to talk to people’. There was evidence from all stakeholders that the peer-supporters had given peer support as intended, including sharing knowledge with friends and offering to co-participate in clubs, sports, incidental physical activity and active play. The peer-supporters reported being empathetic to their friends and trying to encourage them to try new activities and persist with those in which they are already engaged:

*Now it’s [what they suggest doing with friends] do more active things like roller skating or swimming or go out to the park or something instead of just sitting inside.*

*School 4, Peer-supporter focus group*

*Whereas before they go to a youth club and I used to drop them off, now she’s walking. She gets her friends to call, they call for each other and they walk.*

*School 3, Parent of peer-supporter*

*I didn’t realise what [peer-supporter] said when she said they make them walk around and actually I have seen them a while ago on the field and playing on the field [ … ]*

*School 6, School contact*

Peer supporters carefully considered their diffusion attempts:

*I tried to incorporate something that they liked. Erm, cos some of my friends like to dance so it’s finding something that they like and encouraging them to do that more.*

*School 6, Peer-supporter focus group*

Being a peer-supporter was ‘*quite cool*’ *(School 4, Peer-supporter focus group)*. Parents confirmed that their daughters ‘*loved it*’ *(School 2, Parent of peer-supporter)* and did ‘*remarkably well*’ *(School 4, Parent of peer-supporter)* in their role. Peer-supporters faced some challenges when carrying out their role, these were either fear of potential adverse reactions of their friends to peer support (i.e., offending people or being met with hostility), difficulties in starting conversations or having limited friends outside of the peer-supporter group.

*[ … ] she [a peer-supporter] pointed around the room and she was like “Yeah, but these are all of my friends. I don’t have any other friends. All my friends are right here.”*

*Trainer B, Top-up*

Most non-peer-supporters were accepting of support given to them, some taking longer than others to be open to it.

*At first, yeah [non-peer supporter] was very negative about it, but over time she changed a lot - her mind [ … ] so we don’t want to give up on her because she does have potential, I think.*

*School 2, Peer-supporter focus group*

Some non-peer-supporters felt that they had not received any peer-support.

*No, no-one said anything to me.*

*School 4, Non-peer-supporter focus group*

However, peer-supporters believed that this may be due to the informal way they gave support:

*They didn’t really know I was peer supporting because with some friends we did it quite subtly.*

*School 2, Peer-supporter focus group*

During the top-up day training, trainers were initially uncertain as to whether some peer-supporters were being truthful about their efforts:

*Some were like clearly covering up that they hadn’t done it and saying they had.*

*Trainer B, top-up*

### Fidelity to the intervention theories

With regards to DOI, peer-supporters demonstrated that they understood the potential effect of support from close peers and were confident to use informal and supportive (vs. forceful) strategies to diffuse information and support their peer’s physical activity:

*When it’s coming from a friend or someone that they’re close with then they’re more sort of open about being active and what they would like to do, maybe rather than … So I feel that it’s sort of better coming from a friend.*

*School 4, Peer-supporter focus group*

*There are some people (for) who(m) (you) can’t make it obvious that you are trying to encourage them to be fit or physically active, you have just got to like every now and then just say something like “Do you want to walk to school with me?”*

*School 2, Peer-supporter focus group*

Trainers agreed that the SDT-based principles behind PLAN-A were well explained and interesting, however suggested a recap and the end of the training. Whilst the approach was different to the previous motivational style used by one trainer, most found that it aligned with their usual approach to working with young people.

*I think girls particularly need that encouragement and support and that motivational talk and confidence building, I think yeah they really can benefit from it.*

*Trainer A*

Peer-supporters rated the trainers’ interpersonal style as highly autonomy-supportive throughout the two-day training (Mean ± SD = 3.31 ± 0.63) and slightly lower for the top-up day (2.92 ± 0.80). The interviews, focus groups and observations supported these findings indicating that trainers provided, and peer-supporters experienced support for autonomy, competence and relatedness. The trainers made efforts to provide choice, valued the peer-supporters input, used autonomy-supportive language, regularly provided positive feedback and used role models to facilitate learning.

*In (school) classes we just get a teacher telling us things, we were a lot more involved in what was going to happen and things like that.*

*School 4, Peer-supporter focus group*

*They understood. They were saying it nicely, not telling us like do that, do this*

*School 3, Peer-supporter focus group*

Autonomy support was more difficult when the peer-supporters’ behaviour was challenging (i.e., poor listening and engagement, mainly in one school), and trainers attempted to motivate peer-supporters to engage through encouragement.

*We were quite concerned that the amount of times we had to say to them “okay, it’s time to be quiet now”[ … ] they were going to end up not liking us and the not liking us would impact on how positive they felt about the study, about being a peer-supporter. So we were really trying to combat that on the second day, to be really encouraging.*

*Trainer E*

Trainers supported competence by making efforts to ensure that the peer-supporters understood their role, that they knew how to incorporate facts into conversations and were realistic with their attempts to provide support. They also recognised when the peer-supporters were struggling and would break down or add activities to help. Trainers felt that peer-supporters understood what they were teaching:

*The way that they were genuinely understanding what we we’re talking about and saying examples and it was amazing that what we were saying to them was getting through to them.*

*Trainer B*

Peer-supporters felt confident to use the informal approach recommended:

*Like slipping it into a conversation was pretty easy after we learnt how to do it [agreement]. Like before you’d be like ‘urrrr’ [ … ] But, yeah, learning it I think it actually helped.*

*School 4, Peer-supporter focus group*

Despite this, observations noted that support for competence was weaker where trainers gave vague answers to questions and did not always address errors in girls' understanding. Trainers also focussed quite heavily on having conversations as a method of peer-supporting and less on other methods (i.e. co-participation).

There was strong qualitative evidence to suggest that the trainers provided support for relatedness and formed a rapport with the peer-supporters. Specifically, they listened to and valued the girls’ opinions, talked about their life outside of PLAN-A, circulated around groups offering help and joined in with activities. Peer-supporters described them as *‘not like teachers’* in that they were perceived to be more understanding and approachable.

*They were telling us a bit about themselves because we told them a bit about ourselves.*

*School 3, Peer-supporter focus group*

The trainers empathised with and felt trusted by peer-supporters to discuss difficulties that they were experiencing outside of PLAN-A.

*I think it was nice that they could probably talk to us and they know that we, you know, they could trust us. We wouldn’t go in and talk about them behind their back and I think, and I could trust them as well.*

*Trainer C*

### Perceived intervention effects

Parents commonly noted improvements in their daughter’s confidence following PLAN-A, resulting in confidence to voice their opinions at school, trying new things and talking to different people. Peer-supporters and school contacts agreed.

*I think it’s [PLAN-A] empowered her even more to take charge of situations. [ … ] I definitely think it did because she does like to try and get other people involved in things.*

*School 6, Parent of peer-supporter*

*I think they’ve grown in confidence and probably their communication skills.*

*School 4, School contact*

In one school, some non-peer-supporters described peer-supporters as ‘a bit more boastful’ *(School 3, non-peer-supporter focus group)* and that ‘*they get a bit big-headed, just ‘cause they did get chosen’ (School 3, non-peer-supporter focus group)*.

Peer-supporters stated that they were more aware of their own physical activity levels and were more active*.* There was evidence from the spectrum of stakeholders for other potential positive effects of PLAN-A including participation in extracurricular activities, physical activity knowledge, use of positive motivational strategies and relationships between Year 8 girls in school.

*It has helped me become a better person in how I talk to people as well, like making me think before I say to some people like I know that before I probably, if we didn’t have the PLAN-A thing, I probably would have just gone to someone ‘oh you need to be active, come on a run’*

*School 3, Peer-supporter focus group*

Key messages around physical activity and gender inequalities were recalled by some girls demonstrating potential changes in perceptions:

*Everybody used to say that [‘Like a girl’ expression], it [a video] just like put a different perspective in your mind, like everybody runs the same, it’s not like a different way of running for a girl than it is a boy [ … ] I never even thought about it until I … [did the training]*

*School 6, Peer-supporter focus group*

*What I quite often hear at the moment after that [Peer-supporter training] is the girls will say to each other ‘For goodness sake woman up’.*

*School 2, School contact*

In some cases, perceived effects could not be solely linked to PLAN-A.

*In my group, a lot of them did actually start going to the gym now but I don’t know if it was ‘Your allowed to go to the gym now let’s go for it’ or if it was ‘We should do this because of PLAN-A’.*

*School 2, Non-peer-supporter focus group*

### Intervention refinements

The process evaluation revealed several intervention refinements that could be made should the study proceed to a further trial. Trainers felt it would be helpful for the key SDT principles to be covered more than once throughout the train-the-trainers to reinforce their delivery in practice. Other refinements related to the peer-supporter training. It was important for the peer-supporters to have outdoor / breakout space to aid engagement and focus, this should be considered when choosing venues for the training. Delivery was challenging when the peer-supporters lost focus and engagement. Modifications to the training timetable (activities requiring greater concentration in the morning), or the activities themselves (e.g., more active or involving more team work) may resolve this issue. Trainers suggested changes to individual activities (e.g., giving clarity on group sizes for tasks, adding more team work, giving alternative methods to achieve learning objectives where engagement is low, & simplifying some terminology). Other changes to peer-supporter training content included providing more information on how to overcome challenges as a peer-supporter and having more specific examples of how to provide support and who to. These refinements are all achievable within the current overarching intervention structure.

## Discussion

We have previously reported that the PLAN-A intervention shows good school, participant, trainer and peer-supporter recruitment and retention, high levels of data provision, high attendance at peer-supporter training and that the intervention could positively affect adolescent girls’ MVPA [14]. The results presented in this paper expand on these findings with qualitative and quantitative process evaluation data on intervention acceptability, fidelity, receipt, potential impact and potential intervention refinements.

The peer nomination process was acceptable and identified individuals who took pride in their role which echoes findings from the ASSIST study [21]. The peer selection approach in PLAN-A is different from most peer-based physical activity interventions where they follow school year group hierarchies (i.e., older students mentor younger students) [22–25]. The MOVE project [23] recently used this model in a cluster RCT involving 1495 young people in 60 English schools but showed no effect of the intervention on accelerometer-assessed physical activity. In PLAN-A, the use of key influencers from within peer groups is based on a hypothesis that they are sufficiently similar (i.e., in age, interests, life and educational stage, context and priorities) to understand and influence their close friends. However, an unexpected finding was that peer nomination (and potential influence) can be affected by the structure of year groups in schools (e.g., year groups split in two halves which are relatively separate in terms of teaching and other timetabled activities). Interventions using peer nomination / selection should consider implementing peer nomination in ways that take account of the year group structure.

The train-the-trainers course and supporting materials were well-received. The peer-supporter training was delivered with high fidelity to the training manual and the underpinning theory [26], and there was qualitative and quantitative evidence that trainers supported the peer-supporters’ autonomy, competence and relatedness. Relatedness was particularly strong and a respectful, trusting rapport was built which facilitated peer-supporter engagement. The findings reflect previously-identified teacher behaviours which support relatedness in Physical Education settings [27]. In line with previous research [28–30] this suggests that it is possible to train intervention deliverers to use an autonomy-supportive style. Interactive, discussion-led and active learning worked best and was most enjoyed. Challenges to fidelity in delivery were posed when pupils’ behaviour was disruptive, their engagement was low and when activities involved excessive writing or individual work. Previous school-based physical activity interventions based on SDT and the ASSIST implementation [31] have identified similar challenges (i.e., delivering in an autonomy-supportive style when facing disruptive behaviour) [28, 32]. Whilst the train-the-trainers’ workshops incorporated sections on managing behaviour and provided advice on the use of structure and agreed ground rules, trainers requested and need greater practical guidance on managing disruptive behaviour. The school that could not support off-site peer-supporter training held training within the school facility, which provided an opportunity to test an alternate delivery location. Overall, within school delivery was possible and had some logistical advantages, however the potential for disruption from other pupils and usual school activities and constraints by school day timings suggest that off-site delivery would be preferable. The reason for in-school delivery was one school not being able to provide staff time to chaperone the peer-supporters off site, therefore an additional payment to schools to cover the cost of a chaperone might be needed.

Peer-supporters reported enjoying their role and used a variety of strategies to provide peer-support (e.g., co-participation, sharing information, encouragement). They also reported using autonomy-supportive approaches, carefully considering how they encouraged their peers to be active, using empathy and subtlety. Similar to ASSIST [31], some peer-supporters struggled to initiate conversations. Whilst the peer-supporter training could provide more practical advice and role-play on this, the strength of the findings with regards to peer-supporters being empathic and subtle, points towards the importance of promoting less overt, more subtle support strategies. These may fit more naturally into usual peer-peer exchanges and giving social support for being active (e.g., *“Do you want to walk to school with me?”*) and could overcome barriers of low confidence and fear of embarrassment related to the more direct sharing of facts.

Echoing findings from the implementation of ASSIST [31], stakeholders identified a range of perceived positive outcomes on pupils who participated in PLAN-A. Interestingly, many of these were not limited to physical activity levels and knowledge, but included increased confidence, positive self-perceptions, communication skills and challenges to gender-biases (e.g., ‘*For goodness sake woman up’*).

The process evaluation identified a number of refinements that could be made to the PLAN-A intervention before it is tested further. These included revisiting the SDT principles at the end of the train-the-trainers programme, carefully considering the space used for peer-supporter training, refining activities to ensure peer-supporter engagement and revisions to simplify some activities and materials. All are achievable refinements within the current intervention design.

### Strengths and limitations

The mixed methods approach employed allowed us to thoroughly investigate the process within the PLAN-A intervention and triangulate sources of evidence which provides confidence in our findings. The methods and findings are reported in accordance with the COREQ guidance. The perspectives of multiple stakeholders from all schools were gathered, including pupils selected for focus groups from across the spectrum of physical activity levels. Further, data analysis was undertaken prior to and independently from the analysis of the feasibility trial outcomes [14]. However, it is also important to note that due to staff and budget limitations, whilst the process evaluation was conducted by experienced members of the project team they were not external evaluators. We did not interview the parents of non-peer-supporters who, whilst potentially not having good knowledge of the intervention, may have offered further insight into their child’s perspective on the peer nomination process and not being selected. Whilst developing the questionnaires that were used to evaluate key components of the PLAN-A intervention provides specificity, a limitation of this approach is the lack of evidence for reliability or validity of the scores that such scales generate. Further, despite the in-depth process evaluation, the external validity of the findings is limited as the research was a feasibility study and therefore the sample included only four intervention schools and five trainers. Further research is needed to understand implementation and fidelity in a wider range of schools and trainers.

## Conclusion

PLAN-A is an acceptable school-based physical activity intervention for adolescent girls, which can be delivered with high fidelity to both the content and underpinning theories. Some minor refinements would improve on identified challenges to delivery and implementation prior to further testing in a larger trial.

## Data Availability

The questionnaires, focus group and interview guides and datasets generated and/or analysed during the current study are available in the University of Bristol Research Data Storage Facility repository [https://data.bris.ac.uk/data].

## Abbreviations

ASSIST: A Stop Smoking In Schools Trial
COREQ: COnsolidated criteria for REporting Qualitative research
DOI: Diffusion of innovations
MVPA: Moderate-to-vigorous physical activity
PLAN-A: Peer-Led physical Activity iNtervention for Adolescent girls.
RCT: Randomised Controlled Trial
SDT: Self-determination theory
